# Studies on bioflocculant production by a mixed culture of *Methylobacterium* sp. Obi and *Actinobacterium* sp. Mayor

**DOI:** 10.1186/1472-6750-13-62

**Published:** 2013-08-01

**Authors:** Ntsaluba Luvuyo, Uchechukwu U Nwodo, Leonard V Mabinya, Anthony I Okoh

**Affiliations:** 1 Applied and Environmental Microbiology Research Group (AEMREG), Department of Biochemistry and Microbiology, University of Fort Hare, Private Bag X1314, Alice 5700, South Africa

**Keywords:** Bioflocculant, Consortium, Flocculating activity, Thermostable, Functional groups

## Abstract

**Background:**

Bioflocculants effect the aggregation of suspended solutes in solutions thus, a viable alternative to inorganic poly-ionic and synthetic organic flocculants which are associated with deleterious health problems. Consequently, a consortium of two bacteria species were evaluated for optimized bioflocculant yield following the inadequacies of axenic cultures.

**Results:**

16S rDNA nucleotide sequencing and BLAST analysis of nucleotide sequences were used to identify the bacterial species, carbon and nitrogen sources optimally supporting bioflocculant production were assessed and the purified bioflocculant characterized.

Nucleotide sequences showed 97% and 96% similarity to *Methylobacterium* sp. AKB-2008-KU9 and *Methylobacterium* sp. strain 440. The second isolate, likewise, showed 98% similarity to Actinobacterium OR-221. The sequences were deposited in GenBank as *Methylobacterium* sp. Obi [accession number HQ537130] and *Actinobacterium* sp. Mayor [accession number JF799090]. Flocculating activity of 95% was obtained in the presence of Ca^2+^ and heat-stability was exhibited with retention of above 70% activity at 100°C in 30 min. In addition, bioflocculant yield was about 8.203 g/l. A dose of 1 mg/ml of purified bioflocculant was optimal for the clarification of Kaolin suspension (100 ml) following Jar test. FTIR spectrum revealed the presence of carboxyl and hydroxyl functional groups amongst others.

**Conclusions:**

The mixed culture produced bioflocculant with high flocculating activity and an improved yield. The efficiency observed with jar test may imply industrial applicability.

## Background

Flocculants may be synthetic or natural in origin. However, they lead to the fluffy mass formation of suspended particles [1]. Flocculants are extensively applied in the treatment of wastewaters and other industrial effluents [2,3]. Other applications have included the recovery of suspended solutes from solutions [4]. Nonetheless, inorganic flocculants which includes the salts of poly-aluminium chloride and aluminium sulphate as well as the organic synthetic flocculants (poly-acryl amide and polyethylene amine) have been implicated in various human health problems such as nuerotoxicity, cancer and a medical disorder leading to dementia (Alzheimer’s disease). The organic synthetic flocculants are also known to be non biodegradable hence, not environmentally friendly [5]. In contrast, bioflocculants have not been associated with any medical problem and are biodegradable; as such, are considered environmentally friendly [6,7].

Considerable attention has been directed towards studying bioflocculant producing bacteria in axenic culture and yield optimization has been attempted through the manipulation of fermentation and nutritional conditions. Following the aforementioned techniques, high flocculation activities have been documented. However, low bioflocculant yield and lack of cost effectiveness in the production of bioflocculant militates against the application of these bioflocculants in industrial processes, such as in wastewater treatment [8,9].

Consequently, it has become imperative to explore alternative means of bioflocculant yield optimization [10-12]. The application of mixed culture in the production of bioflocculant has been attempted by Kurane and Matsuyama [3] as well as Zhang et al. [13] and bioflocculant yield was reported to have improved. Following these findings, we evaluated the bioflocculant production potentials of a consortium of two fresh water bacteria belonging to *Methylobacterium* and *Actinobacterium* genera and the bioflocculant was characterized for novelty.

## Methods

### Bacterial strains

Bacterial strains were previous isolates from the Tyume River in the Eastern Cape Province of South Africa. Isolates were preserved in glycerol at −80°C as part of the culture collection of the Applied and Environmental Microbiology Research Group (AEMREG), University of Fort Hare, South Africa. However, prior to storage, the test bacteria were identified as *Actinobacterium* sp. Mayor and *Methylobacterium* sp. Obi through partial nucleotide sequencing of their 16S rRNA genes with subsequent BLAST analyses. Nucleotide sequences were deposited in GenBank and the repository accession numbers were JF799090 and HQ537130 respectively.

### Mixed culture fermentation for bioflocculant production

*Actinobacterium* sp. and *Methylobacterium* sp. were activated by inoculation of 20 μL of the glycerol stock into a sterile 5 mL broth composed of (g/L); beef extract (3), tryptone (10) and NaCl (5) and each was incubated overnight at 28°C respectively. One percent (1%), each, of the activated culture was inoculated into 400 ml of bioflocculant production medium in 1000 ml conical flask. Bioflocculant production media was prepared in accordance with the methods of Zhang et al. [13]. Briefly, glucose (20.0 g), KH_2_PO_4_ (2.0 g), K_2_HPO_4_ (5.0 g), (NH_4_)_2_SO_4_ (0.2 g), NaCl (0.1 g), MgSO_4_ · 7H_2_O urea (0.5 g) (0.2 g) and yeast extract (0.5 g) were dissolved in one litre of distilled water and the pH adjusted to 7. The incubation conditions for the mixed culture fermentation were an incubation temperature of 28°C, agitation speed of 160 rpm in a shaker incubator and fermentation time of 72 h. Thereafter, the fermentation broth was centrifuged at 3000 rpm for 30 min at 15°C and the cell-free supernatant was assayed for flocculation activity.

### Effect of inoculum size and pH on bioflocculant production

Mixed culture inoculum volumes of 0.5%, 1%, 1.5% and 2% in proportion to the fermentation volume (400 ml) were respectively evaluated for bioflocculant production. The cultures were incubated at a temperature of 28°C for 72 h at 160 rpm. Thereafter, the fermentation broth was centrifuged (3000 rpm, 30 min, 15°C) and the supernatant was assessed for flocculation activity. Likewise, the initial fermentation pH regimes of 2 to 12 were evaluated for bioflocculant production while other conditions were kept constant.

### Flocculation activity determination

Flocculating activity was determined in accordance with the methods of Kurane et al. [8] as modified by Wang et al. [14]. A suspension of Kaolin clay (4 g/L) in deionized water at pH 7.0 was used. One hundred micro liters (100 μL) of the bioflocculant-rich broth and 250 μL of 1% CaCl_2_ were added to 900 μL of the Kaolin clay suspension; the mixture was votexed at 50 rpm for 60 sec and allowed to stand for 5 min at room temperature. Bioflocculant-rich broth was replaced with deionized water as a control and optical densities (OD) of the clarifying upper phase of the solution was measured at 550 nm using a ThermoSpectronic spectrophotometer (Helios Epsilon, USA). Flocculating activity was determined as follows:

$$\mathrm{Flocculating}\;\mathrm{activity}=\left[\left(B-A\right)/B\right]\times 100\%$$

*A* and *B* were optical densities at 550 nm of the sample and control respectively.

### Purification of bioflocculant

The concentration and purification of bioflocculant from the bioflocculant-rich broth was in accordance with the methods of Chang et al. [15]. One volume of distilled water was added to the cell-free-bioflocculant-rich broth and centrifuged at 10 000 rpm for 15 min at 15°C, the supernatant was decanted and the residue re-suspended with 20 ml of distilled water. Two volumes of cold ethanol were added to the bioflocculant solution and the mixture was left standing at 4°C for 12 h. after which the precipitate was collected through centrifugation (10 000 rpm; 15 min; 15°C). The residue was washed twice with distilled water, lyophilized and vacuum dried. The dried bioflocculant was used for subsequent assays.

### Optimum bioflocculant concentration for flocculation activity – Jar test

In accordance with the methods of Wang et al. [14], Jar-test was employed, with some modification, to determine bioflocculant concentration optimally mediating flocculation of Kaolin clay suspension (4.0 g/L). Bioflocculant concentrations of (mg/ml); 0.5, 1.0, 1.5 and 2.0 were respectively added to 100 ml Kaolin clay suspension (4.0 g/L) containing 3 ml of 1% CaCl_2_ in 500 ml beakers. The mixture was rapidly stirred at 180 rpm for 3 min, followed by slow stirring at 40 rpm for 5 min. The solutions were then allowed to stand for 10 min. and afterwards, flocculating activity was measured and calculated as previously described.

### Effect of temperature, pH and cations on flocculating activity

The effect of temperature regimes on the flocculating activity of purified bioflocculant were investigated; desired concentration of purified bioflocculant was reconstituted with 10 ml of distilled water and incubated in water bath at the respective temperatures; 50°C, 80°C and 100°C for a period of up to 30 min. Residual flocculating activity were measured afterwards [16]. Similarly, the effect of pH on flocculation activity of bioflocculant was determined by adjusting the pH of Kaolin clay suspension from 3 to 12 using HCl or NaOH, before the addition of bioflocculant and CaCl_2_ as previously described. Furthermore, KCl, NaCl, LiCl, MgCl_2_, MnCl_2_, AlCl_3_ and FeCl_3_ were respectively assessed as cation sources in place of CaCl_2_[10], all conditions for flocculation activity assay were kept constant.

### FT-IR spectroscopy and thermo-gravimetric analyses of purified bioflocculant

The functional groups of the bioflocculant were determined using Fourier transform infrared spectrophotometer (Perkin Elmer System 2000, FT-IR, England). The bioflocculant was ground with KBr at room temperature and pressed into a thin disc for FTIR spectroscopy over a wave number range of 4 000 - 370 cm^-1^. The thermo-gravimetric analysis of the purified bioflocculant was carried out at the temperature range of 20 to 900°C with a heating rate of 10°C/min under a constant flow of nitrogen gas, using a thermogravimetric analyzer (TGA 7; Perkin Elmer) fitted with thermal analysis controller (TAC 7/DX).

## Results and discussion

### Effect of inoculum size on bioflocculant production

**Figure 1:**
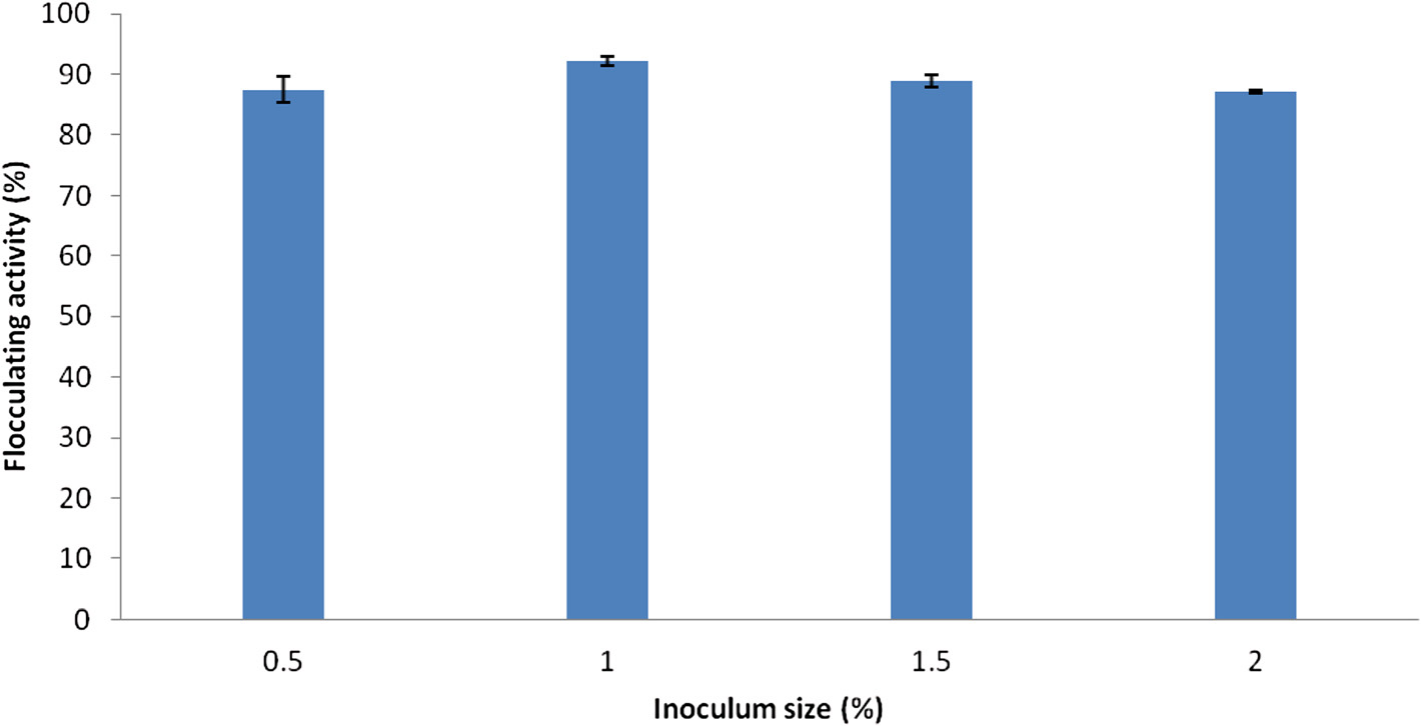
**Effect of the inoculum size of *Actinobacterium *sp. and *Methylobacterium *sp. consortium on bioflocculant production.**

An optimum flocculation activity of 92% was achieved with 1% (v/v) of the mixed culture (Figure 1). Higher inoculum cell densities (1.5% and 2.0%) evaluated, did not yield corresponding increase in flocculating activity. Rather, a slight decrease was observed. This observation is expected as inappropriate ratio of inoculum cell densities to nutrient ratio leads to a phenomenon termed “inoculum effect” consequently, a reduction in the desired effect. Similar trend was observed by Zhang et al*.*[13] in a study on bioflocculant production by a consortium of *Staphylococcus* and *Pseudomonas* species, while Wang et al*.*[17] obtained maximum flocculating activity for a bioflocculant produced by an axenic culture of *Klebsiella mobilis* when 5% inoculum size was used.

### Effect of pH on bioflocculant production

**Figure 2:**
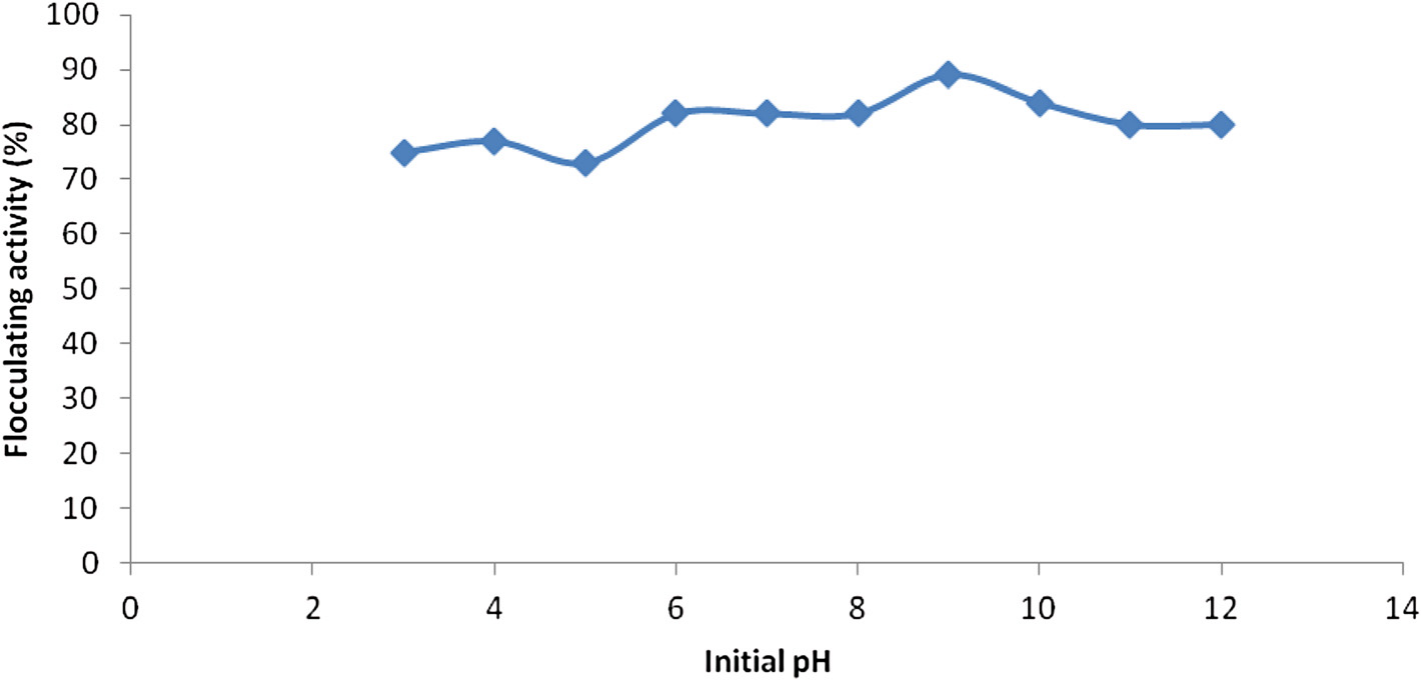
**Effect of initial fermentation pH on bioflocculant production by the consortium of *Actinobacterium *sp. and *Methylobacterium *sp.**

An alkaline pH of 9, at the start of fermentation, optimally supported bioflocculant production with flocculation activity of 89% (Figure 2). At acidic initial fermentation pH, lower flocculation activity was observed. In addition, fermentation time was prolonged yet, comparable flocculation activity with the ambient pH was not achieved. The longer time observed at acidic initial fermentation pH may be interpreted as time needed for the mixed culture to adjust physiologically to the acidic medium. Nonetheless, various initial medium pH has been reported for the production of bioflocculant by different microbial species. Wang et al*.*[14], reported optimum bioflocculant production by *Rhizobium radiobacter* F2 and *Bacillus sphaeicus* F6 at neutral and weak alkaline pH while Nwodo et al. [18] reported weak acidic condition (pH 6.8) as optimally supporting bioflocculant production by *Streptomyces* sp. Gansen.

### Bioflocculant yield and flocculation of kaolin clay

Mixed culture fermentation, following optimal conditions (starter culture density of 1%, initial fermentation pH of 9, agitation speed of 160 rpm and incubation temperature of 28°C), yielded bioflocculant to the tune of 8.203 g/l after purification. Similar account was documented by Zhang et al. [13]. However, the yield with mixed cultures of *Methylobacterium* sp. Obi and *Actinobacterium* sp. Mayor reported in this work was lower than those from the consortium of *Staphylococcus* and *Pseudomonas* species [13].

**Figure 3:**
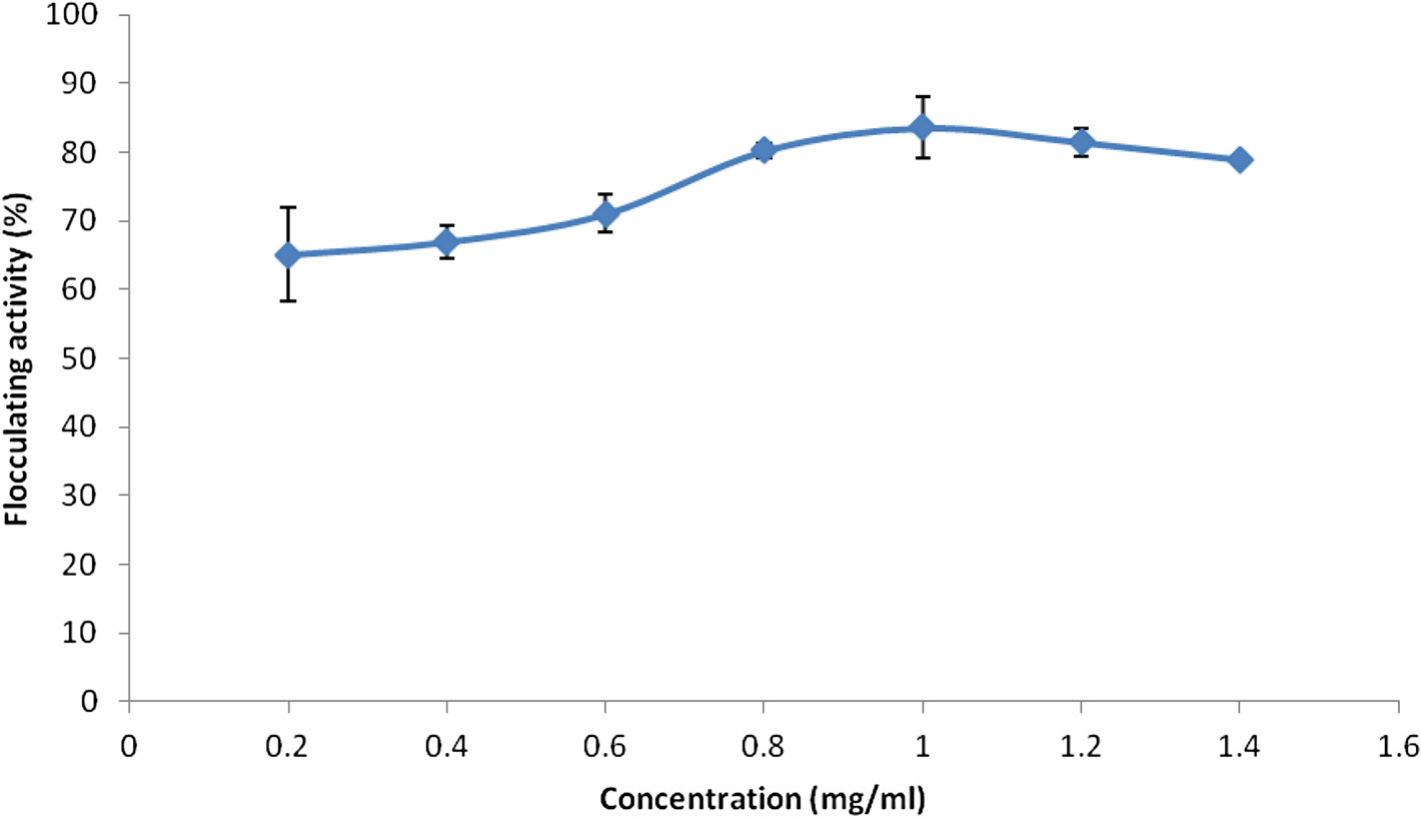
**Optimum flocculating activity determination of purified bioflocculant produced by *Actinobacterium *sp. and *Methylobacterium *sp. consortium following Jar-test.**

The evaluation of bioflocculant concentrations, optimal, for flocculation activity revealed 1 mg/ml in 100 ml Kaolin clay suspension (4 g/L) following the Jar test experimentation (Figure 3). At higher concentrations of bioflocculant, flocculation activity declined and this may be explained as inappropriate interaction of the surfaces charges on the bioflocculant in the medium due to saturation effect consequently, less surface area for binding activity ensured. Better still, the counteractive effect of higher bioflocculant concentration, which disturbs the surface charge distribution, may have accounted for the observation. On a similar note, Chan and Chiang, [19] observed that, when flocculants optimum concentration required for flocculation is exceeded; aggregated particles are re-dispersed thus, disallowing particle settling. Nonetheless, optimum concentrations required for flocculation activity are organism dependent; Wang et al*.*[20] reported 12 mg/l, from the bioflocculant produced by the consortium of *Rhizobium radiobacter* F2 and *Bacillus sphaeicus* F6, as the optimum concentration required for the flocculation of Kaolin clay in Jar test experimentation. Likewise, Zheng et al. [21] reported 80 mg/l of bioflocculant produced by *Bacillus* sp. as optimally causing flocculation of Kaolin clay. Conversely, a lower concentration of 0.3-8.2 (mg/l) with flocculation activity at above 90% [22] was reported for *Chryseobacterium daeguese* W6.

### Flocculation activity of purified bioflocculant - effects of physical-chemical factors

**Figure 4:**
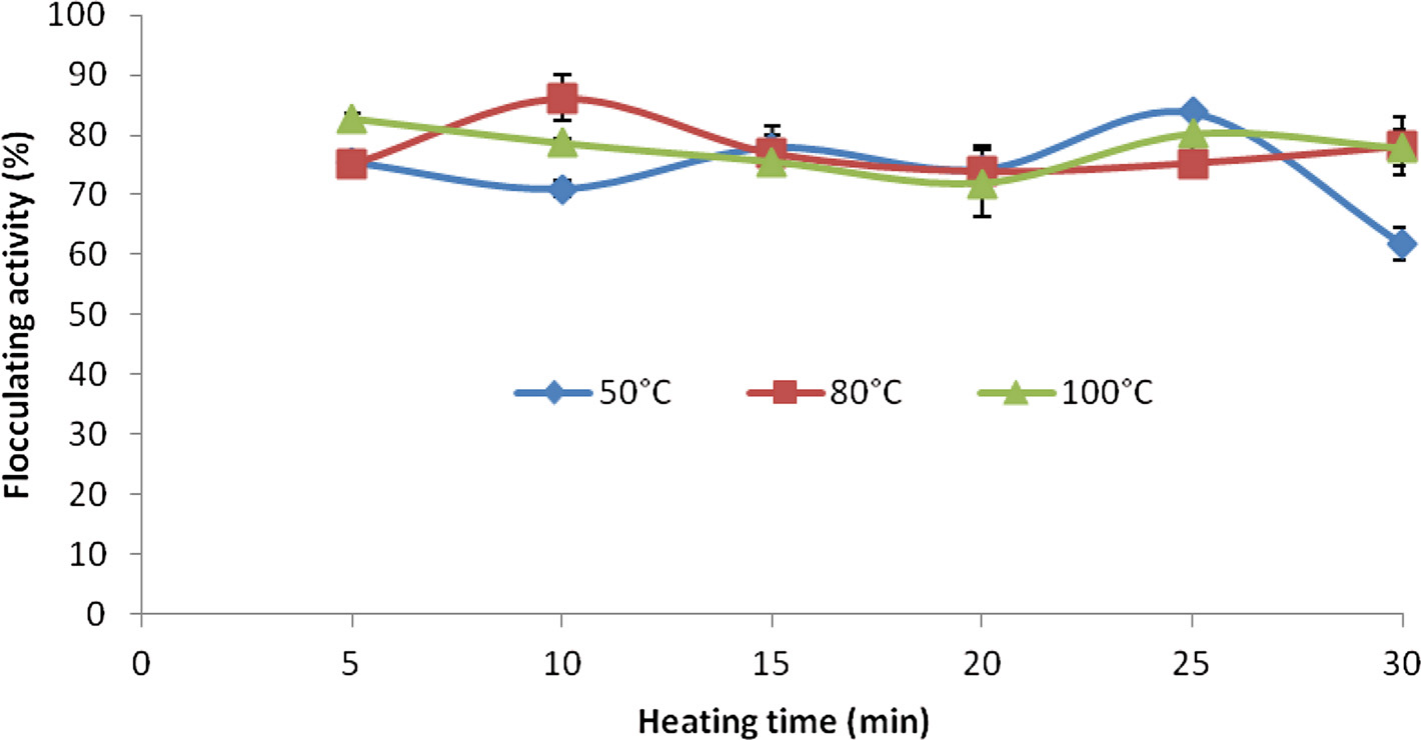
**Stability of the purified bioflocculant produced by *Actinobacterium *sp. and *Methylobacterium *sp. consortium to different medium temperature.**

Subjecting the bioflocculant to different temperature regimes (50°C, 80°C and 100°C) for a period of 30 min showed thermal stability as flocculation activity was retained. At 80°C, flocculation activity of 86% was obtained (Figure 4). The heat stability of the bioflocculant is remarkable as flocculation activity of more than 70% was retained at 80°C and 100°C of the incubation time. The bioflocculant produced by *Rhizobium radiobacter* F2 and *Bacillus sphaeicus* F6 [19] consortium was similarly reported to exhibit thermal stability. Temperature tolerance of polymers is important in deciding suitability for water treatment Patil et al. [4].

**Figure 5:**
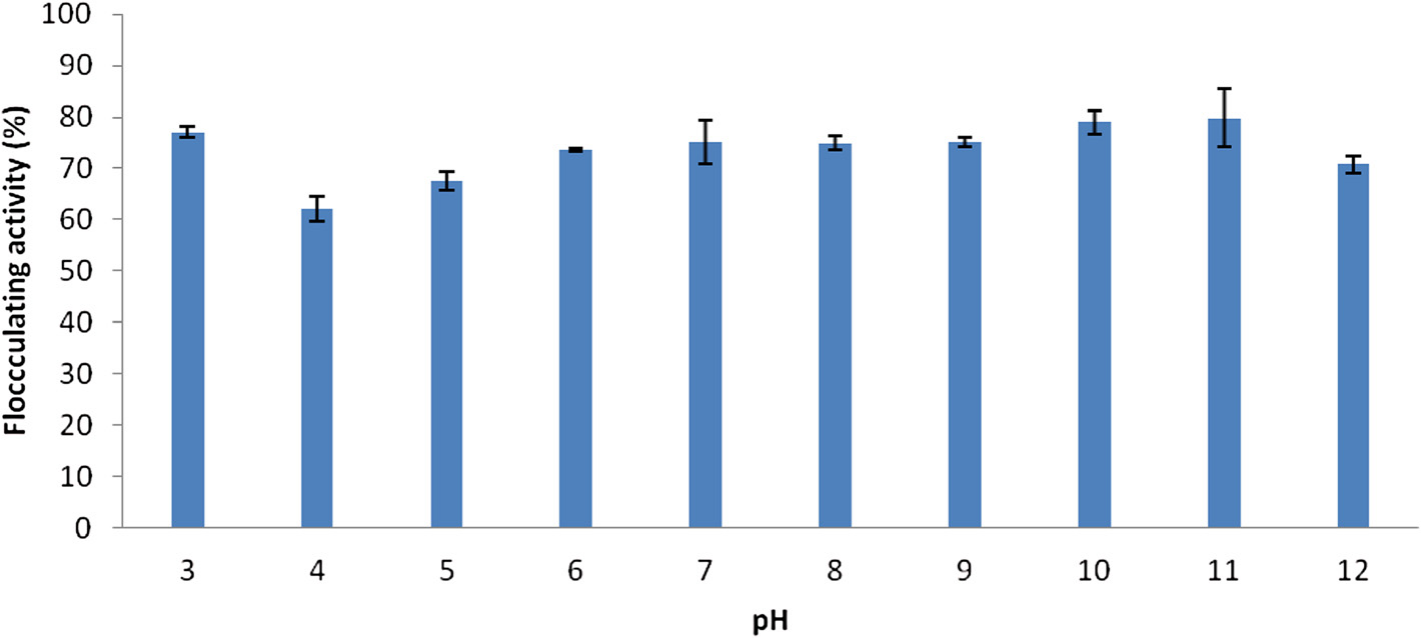
Effect of pH on flocculating activity of purified bioflocculant.

Adjusting the pH of the Kaolin clay suspension from acidic to alkaline (3-12) showed recalcitrance to the effects of H^+^ concentration. Flocculating activities of more than 60% were observed in the pH range assessed with the highest flocculating activity of 79% obtained at pH 11. Similarly, high flocculation activity (76%) was observed at the acidic pH of 3 (Figure 5). The reason behind the dual flocculation optimum, at an acidic and alkaline pH is unclear. However, the weak acidic pH of 6 was reported as optimum for the bioflocculant produced by the mixed cultures of *Staphylococcus* and *Pseudomonas*[13]. Nwodo et al. [18] reported neutral pH as optimally supporting flocculation activity of purified bioflocculant. Various pH has been documented to maximally support flocculation activity of bioflocculants [10,20,23].

**Figure 6:**
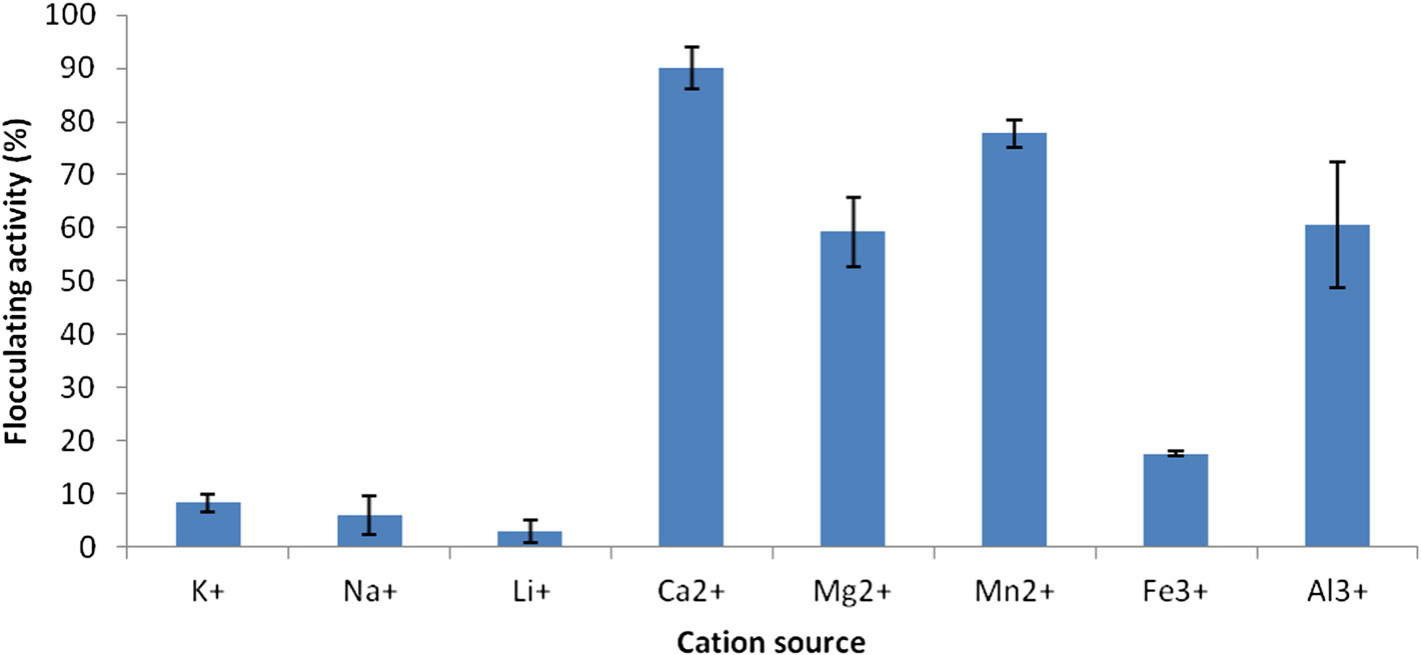
Effect of cation on flocculating activity of purified bioflocculant produced by the consortium.

The assessment of various cations (monovalent, divalent and trivalent) for optimal mediation of flocculation activity of the purified bioflocculant showed the divalent cations of CaCl_2_, MgCl_2_ and MnCl_2_ to best support flocculation activity. The ions of CaCl_2_ were best as flocculating activity of 90% was achieved (Figure 6). Other ion valences have been reported to aid flocculation activity of bioflocculants produced from different microbial species. The bioflocculant produced by the consortium of *Oerskovia, Acinetobacter, Agrobacterium* and *Enterobacter* species was optimally supported by Ca^2+^ in flocculation activity [3]. On the other hand, the trivalent cations of Al^3+^ and Fe^3+^ were reported to be more effective in stimulation flocculating activity of a bioflocculant produced by a consortium of *Rhizobium radiobacter* F2 and *Bacillus sphaeicus* F6 [19]. The support of various ion valences leading to optimal flocculation activity shown by different bioflocculants may be attributed to their surface properties, particularly the distribution of charges on the surface of the biflocculants.

### Compositional analysis of the purified bioflocculant

**Figure 7:**
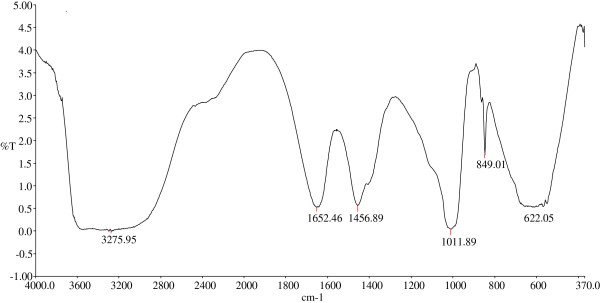
Fourier-transform infrared (FTIR) spectroscopy of purified bioflocculant produced by the consortium.

Fourier-transform infrared (FTIR) spectrum of the pure bioflocculant showed broad absorption band at 3275 cm^-1^ and two other sharp bands at 1652 and 1456 (cm^-1^), which represents the presence of carboxyl and hydroxyl groups from polymeric and dimeric OH stretches of phenol or tertiary alcohol bends [19]. The C-O stretching vibration band at 1011 cm^-1^ in conjunction with sharp peak at 849 cm^-1^ indicates the presence of furan saccharides (Figure 7). Similar spectrum has been reported for bioflocculant produced by other microbial species [7,24,25]. These functional groups provide surface charges which serve as the binding sites for suspended particles hence, causing aggregation or floc formation in solutions and/or colloids. The roles of OH^-^, COO^-^ and H^+^ groups in the flocculation of suspended particles have been reported for several bioflocculants of microbial origin [26,27].

The bioflocculant thermogram revealed an initial weight loss between 20°C and 150°C and afterwards, other decompositions occurred at 590°C, 700°C and 850°C respectively. The thermogram profile indicates generic compounds present in the bioflocculant, with proteins and carbohydrates as an integral constituents.

## Conclusion

The bioflocculant produced by the mixed cultures of *Methylobacterium* sp. Obi and *Actinobacterium* sp. Mayor is composed of proteins and polysaccharides and probably other constituents which have contributed to the high flocculation of Kaolin clay from the solution. In addition, the mixed culture of *Methylobacterium* sp. and *Actinobacterium* sp. have shown good bioflocculant producing potential, following high flocculation activity and bioflocculant yield obtained, in comparison to the yield and flocculation activity shown by the respective axenic cultures. Hence, bioflocculant produced by the consortium has good potentials for industrial applications.

## Competing interest

The authors declare that they have no competing interests.

## Authors’ contributions

NL; Executed the experiment, UUN; extracted the data and drafted the manuscript. LVM; and AIO; designed and supervised the research as well as proof read the final version of the manuscript. All authors read and approved the final manuscript.
